# Steroid hormone receptor expression in ovarian cancer: progesterone receptor B as prognostic marker for patient survival

**DOI:** 10.1186/1471-2407-12-553

**Published:** 2012-11-24

**Authors:** Miriam Lenhard, Lennerová Tereza, Sabine Heublein, Nina Ditsch, Isabelle Himsl, Doris Mayr, Klaus Friese, Udo Jeschke

**Affiliations:** 1 Department of Obstetrics and Gynecology, Grosshadern Campus, Ludwig-Maximilians-University Hospital, Marchioninistrasse 15, Munich, 81377, Germany; 2 Department of Obstetrics and Gynecology, Campus Innenstadt, Ludwig-Maximilians-University Hospital, Maistrasse 11, Munich, 80337, Germany; 3 Department of Pathology, Ludwig-Maximilians-University Hospital, Thalkirchner Str. 36, Munich, 80337, Germany

**Keywords:** Estrogen receptor alpha (ER-α), Estrogen receptor beta (ER-β), Progesterone receptor A (PR-A), Progesterone receptor B (PR-B), Ovarian cancer, Survival, Prognosis

## Abstract

**Background:**

There is partially conflicting evidence on the influence of the steroid hormones estrogen (E) and progesterone (P) on the development of ovarian cancer (OC). The aim of this study was to assess the expression of the receptor isoforms ER-α/-β and PR-A/-B in OC tissue and to analyze its impact on clinical and pathological features and patient outcome.

**Methods:**

155 OC patients were included who had been diagnosed and treated between 1990 and 2002. Patient characteristics, histology and follow-up data were available. ER-α/-β and PR-A/-B expression were determined by immunohistochemistry.

**Results:**

OC tissue was positive for ER-α/-β in 31.4% and 60.1% and PR-A/-B in 36.2% and 33.8%, respectively. We identified significant differences in ER-β expression related to the histological subtype (p=0.041), stage (p=0.002) and grade (p=0.011) as well as PR-A and tumor stage (p=0.03). Interestingly, median receptor expression for ER-α and PR-A/-B was significantly higher in G1 vs. G2 OC. Kaplan Meier analysis revealed a good prognosis for ER-α positive (p=0.039) and PR-B positive (p<0.001) OC. In contrast, ER-β negative OC had a favorable outcome (p=0.049). Besides tumor grade and stage, Cox-regression analysis showed PR-B to be an independent prognostic marker for patient survival (p=0.009, 95% CI 0.251-0.823, HR 0.455).

**Conclusion:**

ER-α/-β and PR-A/-B are frequently expressed in OC with a certain variability relating to histological subtype, grade and stage. Univariate analysis indicated a favorable outcome for ER-α positive and PR-B positive OC, while multivariate analysis showed PR-B to be the only independent prognostic marker for patient survival. In conclusion, ER and PR receptors may be useful targets for a more individualized OC therapy.

## Background

There are various hypotheses to explain the etiology of ovarian cancer (OC), two of them discussing hormonal influence on OC tumorgenesis [1,2]. Until today, the influence of hormones on the development or progression of OC remains under discussion [3,4]. Some hormonal risk factors for the development of OC like nulliparity and infertility have been identified in epidemiologic studies, while pregnancy and oral contraceptives seem to protect from the disease [1,5].

The two steroid hormones estrogen and progesterone act via different hormone receptors. Estrogen (E) and progesterone (P) bind to a nuclear receptor (R), estrogen in addition to an intracellular transmembrane receptor, which is the G-protein-coupled receptor GPR30 [6,7]. In this study we focus on the nuclear receptors ER and PR. Different ER and PR isoforms have been described, ER-α/-β and PR-A/-B, with only slight differences in receptor composition. The PR-A isoform for instance lacks only the N-terminal 164 amino acids of isoform PR-B [8,9]. Their differential regulation of gene transcription might explain their diverse influence on OC progression and prognosis [10]. Progesterone is generally assumed to act antagonistically to oestrogen-mediated cell proliferation [11], though its specific role in OC is unknown. The classic steroid hormone receptors ER-α and PR-A show different effects on OC cells in vitro and in vivo than the recently discovered receptors ER-β and PR-B [12-16].

In contrast to breast cancer or endometrial cancer, where steroid hormone receptor expression is well characterized and known to have therapeutic and prognostic relevance [17,18], there are only few studies with partly contradictory results on OC and ER or PR expression [19,20].

The present study was therefore designed to further analyze ER-α/-β and PR-A/-B expression in a large cohort of OC patients and to assess its impact on clinical and pathological features and patient outcome.

## Methods

### Tissue samples

All tissue samples were gained at surgery in patients who had been operated for primary OC at our institution between 1990 and 2002. Staging and grading were performed by an experienced gynecologic pathologist (D.M.) according to the criteria of the International Federation of Gynaecologists and Obstetricians (FIGO) and the World Health Organization (WHO). Patients with ovarian low malignant potential tumors were excluded from the study. Patient’s clinical data were available from patient charts, aftercare files and tumor registry database information. The main outcomes assessed were disease recurrence and patient survival. For survival analysis, survival time was defined as the time between the date of primary ovarian cancer diagnosis and the date of death.

### Ethics approval

The study has been approved by the local ethics committee of the Ludwig-Maximilians University Munich (approval with the reference number 138/03) and has been carried out in compliance with the guidelines of the Helsinki Declaration of 1975. The study participants gave their written informed consent and samples and clinical information were anonymized for statistical workup.

### Immunohistochemistry

Immunohistochemistry was performed using a combination of pressure cooker heating and the standard streptavidin-biotin-peroxidase complex with the use of the mouse-IgG-Vectastain Elite ABC kit (Vector Laboratories, Burlingame, CA) [21]. Primary antibodies used for immunohistochemical staining were anti-ER-α/-β and anti-PR-A/B.

In short, paraffin-fixed tissue sections were dewaxed with xylol for 15 min, then dehydrated in ascending concentrations of alcohol (70-100%). Afterwards, they were exposed for epitope retrieval for 10 min in a pressure cooker using sodium citrate buffer (pH 6.0) containing 0.1 M citric acid and 0.1 M sodium citrate in distilled water. After cooling, slides were washed in PBS twice. Endogenous peroxidase activity was quenched by dipping in 3% hydrogen peroxide (Merck, Darmstadt, Germany) in methanol for 20 min. Non-specific binding of the primary antibodies was blocked by incubating the sections with “diluted normal serum” (10 ml PBS containing 150 μl horse serum; Vector Laboratories, CA) for 20 min at room temperature. Then, slides were incubated with the primary antibodies at room temperature for 60 min. After washing with PBS, slides were incubated in diluted biotinylated anti-serum secondary antibody (10 ml PBS containing 50 μl horse serum, Vector Laboratories, CA) for 30 min at room temperature. After incubation with the avidin-biotin-peroxidase complex (diluted in 10 ml PBS, Vector Laboratories, CA) for 30 min and repeated PBS washing, visualization was conducted using substrate and chromagen 3,3’-diaminobenzidine (DAB, Dako, Glostrup, Denmark) for 8–10 min. Slides were then counterstained with Mayer’s acidic hematoxylin and dehydrated in ascending concentrations of alcohol (50–98%). After xylol treatment, slides were covered.

Human breast cancer and colon tissue served as positive controls, human ileum as negative. Positive staining appeared in brownish colour, negative as well as unstained tissue blue.

### Immunohistochemical analysis

Slides were evaluated and digitalized with a Zeiss photomicroscope (Axiophot, Axiocam, Zeiss, Jena, Germany). Immunohistochemical staining was assessed using a semiquantitative score according to Remmele and Steger [22], comprising optical staining intensity (graded as 0 = no, 1 = weak, 2 = moderate, and 3 = strong staining) and the percentage of positively stained cells (0 = no, 1 = <10%, 2 = 11–50%, 3 = 51–80% and 4 = >81% cells). The final score is the sum of intensity and percentage scores. According to previously published data, we scored the tumor tissue as positive if more than 10% of cells were scored with an immunoreactive score (IRS) higher than 2 [17,22]. The slides were reviewed in a blinded fashion by two independent observers, including a gynecological pathologist (D.M.).

### Statistical analysis

Statistical analysis was performed using SPSS 18.0 (PASW Statistic, SPSS Inc., IBM, Chicago, IL). Differences in OC receptor expression among three or more groups were tested using the non-parametric Kruskal-Wallis rank-sum test and for pairwise comparisons using the non-parametric Mann–Whitney-U rank-sum test. Correlation analysis was performed using the Spearman correlation coefficient. For the comparison of survival times, Kaplan-Meier curves were drawn. The chi-square statistic of the log-rank test was calculated to test differences between survival curves for significance. Multivariate analysis for prognostic value was performed using the Cox-regression model. P values below 0.05 were considered statistically significant.

## Results

### Patient characteristics

Paraffin embedded tissue of 155 OC patients was available. Median age at primary diagnosis was 59 years (range 21–88). Most patients presented with progressed disease at primary diagnosis [FIGO I: n=35 (22.6%), FIGO II: n=9 (5.8%), FIGO III: n=109 (70.3%), FIGO IV: n=2 (1.3%)]. Patient characteristics are shown detailed in Table 1. Median follow-up time was 12.2 years (95% CI: 9.7-14.6). With 28 documented relapses and 104 deaths, median relapse free survival was 3.7 years (95% CI: 1.9-5.6) and median overall survival 3.4 years (95% CI: 2.2-4.7). Patients with ovarian low malignant potential tumor were excluded from this study.

**Table 1:** OC patient characteristics

		**OC tissue samples**
**OC patients (n)**		155
**Age at primary diagnosis**		59 (range 21–88)
**Median age (a)**		10 (6.7)
**< 40 (n, %)**		23 (15.3)
**40**–**49 (n, %)**		61 (40.7)
**50**–**65 (n, %)**		56 (37.3)
**>65 (n, %)**		
**Histology (%)**	serous	70.5
	mucinous	13.5
	endometrioid	7.7
	clear cell	8.3
**Tumor grade (%)**	low grade	27.2
	intermediate	36.5
	high grade	36.3
**Tumor stage (FIGO) (%)**	I	22.6
	II	5.8
	III	70.3
	IV	1.3
**Gonadotropin receptor expression (%)**[34]	LH-R positive	64.3
	FSH-R positive	63.1
**Oestrogen receptor expression (%)**	ER-α positive	31.4
	ER-β positive	60.1
**Progesterone receptor expression (%)**	PR-A positive	36.2
	PR-B positive	33.8

### Receptor expression in OC tissue

**Figure 1:**
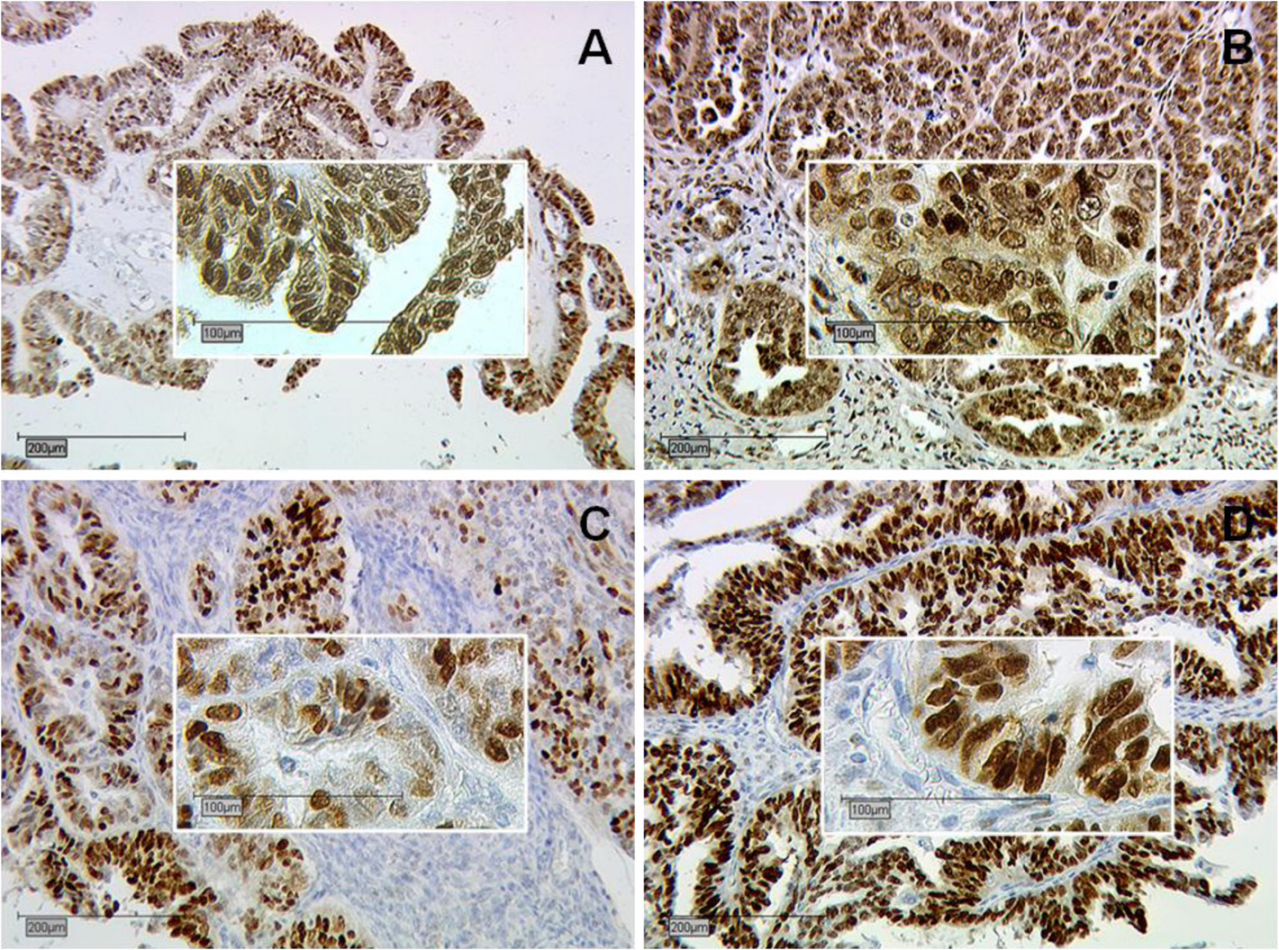
**Representative slides of immunohistochemical staining for ER-α (A, magnification 10x and 25x), ER-β (B, magnification 10x and 25x), PR-A (C, magnification 10x and 25x) and PR-B (D, magnification 10x and 25x) in serous OC.** Immunohistochemical staining was assessed according to the immunoreactive score (IRS) by Remmele and Steger [22]. No receptor immunoreactivity was detected in tumor stroma

OC tissue was positive for ER-α/-β in 31.4% and 60.1% and PR-A/-B in 36.2% and 33.8%, respectively (Table 1 and Figure 1A-D). Highest mean IRS according to the histological subtype was noted for ER-β (mean IRS 3.54±0.259), followed by PR-B (mean IRS 3.44±0.280), PR-A (mean IRS 2.15±0.249) and ER-α (mean IRS 1.90±0.214).

**Figure 2:**
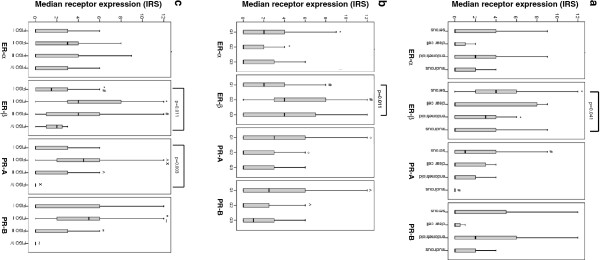
**Median receptor expression for the histological subtype (a), grade (b) and stage (c).a**: Receptor expression and histological subtype. Significant differences were observed for * ER-β: serous vs. endometrioid (p=0.024), # PR-A: serous vs. mucinous (p=0.049) [Mann–Whitney U] and ER-β (all subtypes): p=0.041 [Kryskal Wallis]. **b**: Receptor expression and tumor grade. Significant differences were observed for * ER-α: G1 vs. G2 (p=0.028), # ER-β: G1 vs. G2 (p=0.002), ° PR-A: G1 vs. G2 (p=0.048), ^ PR-B: G1 vs. G2 (p=0.038) [Mann–Whitney U] and ER-β (G1-G3): p=0.011 [Kruskal Wallis]. **c**: Receptor expression and tumor stage. Significant differences were observed for * ER-β: FIGO I vs. II (p=0.005), # ER-β: FIGO I vs. III (p=0.001), ° PR-A: FIGO I vs. II (p=0.019), ^ PR-A: FIGO II vs. III (p=0.017), x PR-A: FIGO II vs. IV (p=0.036), “ PR-B: FIGO II vs. III (p=0.017), ~ PR-B: FIGO II vs. IV (p=0.034) [Mann–Whitney U] and ER-β (FIGO I-IV): p=0.011 and PR-A (FIGO I-IV): p=0.030 [Kruskal Wallis].

The only statistically significant difference in receptor expression of all subtypes was observed for ER-β (p=0.041) (Figure 2a). Pairwise comparison of histological subtypes showed a significant difference for ER-β and the serous vs. endometrioid (p=0.024) and PR-A and the serous vs. mucinous (p=0.049) subtype (Figure 2a).

Significant differences in steroid receptor expression and all tumor grades were observed for ER-β only (p=0.011) (Figure 2b). Interestingly, pairwise comparison showed a significantly higher median receptor expression in G1 vs. G2 for ER-α and PR-A/-B, but inversely low receptor expression in G1 vs. G2 for ER-β tumors [ER-α: G1 vs. G2 (p=0.028), ER-β: G1 vs. G2 (p=0.002), PR-A: G1 vs. G2 (p=0.048), PR-B: G1 vs. G2 (p=0.038)] (Figure 2b).

Comparing all median tumor stages, a significantly different receptor expression was noticed for ER-β (p=0.011) and PR-A (p=0.030). Moreover, pairwise analysis showed statistically significant differences for ER-β in FIGO I vs. II (p=0.005) and I vs. III (p=0.001), for PR-A in FIGO I vs. II (p=0.019), II vs. III (p=0.017) and II vs. IV (p=0.036) and PR-B in FIGO II vs. III (p=0.017) and II vs. IV (p=0.034) (Figure 2c).

### Receptor correlations

We found various positive but no significant negative correlations between ER-α, ER-β, PR-A and PR-B expressions (Table 2). PR-B for instance, which we found to be of prognostic value in this study (see results below), correlates significantly with ER-α (correlation coefficient 0.237, p=0.003) and PR-A (correlation coefficient 0.622, p<0.001) (Table 2).

**Table 2:** Correlation between ER-α, ER-β, PR-A, PR-B, LH-R and FSH-R

	**Correlations**	**ER-α**	**ER-β**	**PR-A**	**PR-B**	**LH-R**	**FSH-R**
**ER-α**	Correlation Coefficient	-	0.058	0.236^**^	0.237^**^	0.045	0.104
	Sig. (2-tailed)	-	NS	**0.003**	**0.003**	NS	NS
	N	-	153	152	154	154	151
**ER-β**	Correlation Coefficient	0.058	-	0.234^**^	0.133	0.032	−0.026
	Sig. (2-tailed)	NS	-	**0.004**	NS	NS	NS
	N	153	-	150	152	151	148
**PR-A**	Correlation Coefficient	0.236^**^	0.234^**^	-	0.622^**^	−0.164^*^	0.069
	Sig. (2-tailed)	**0.003**	**0.004**	-	**<0.001**	**0.045**	NS
	N	152	150	-	152	150	147
**PR-B**	Correlation Coefficient	0.237^**^	0.133	0.622^**^	-	−0.031	−0.060
	Sig. (2-tailed)	**0.003**	NS	**<0.001**	-	NS	NS
	N	154	152	152	-	152	149

** Correlation is significant at the 0.01 level (2-tailed) * Correlation is significant at the 0.05 level (2-tailed) (Spearman’s rho), significant results are shown in bold. NS=not significant.

### Prognostic value

**Figure 3:**
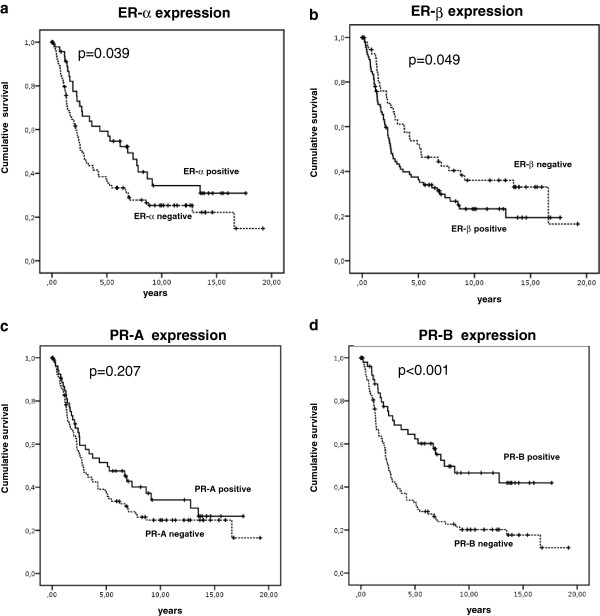
**Kaplan Meier survival analysis for ER-α (a), ER-β (b), PR-A (c) and PR-B (d) receptor expression in OC patients. a**: ER-α positive vs. negative OC. **b**: ER-β positive vs. negative OC. **c**: PR-A positive vs. negative OC. **d**: PR-B positive vs. negative OC,.

Statistical analysis was performed to test for a prognostic value of ER-R or PR-R expression. Univariate Kaplan Meier analysis revealed a good prognosis for ER-α positive (p=0.039) (Figure 3a) and PR-B positive OC (p<0.001) (Figure 3d) OC. Moreover, ER-β negative OC had a favorable outcome (p=0.049) (Figure 3b). A similar trend was observed for the relapse free interval, though not reaching statistical significance (p>0.05). Besides tumor grade and stage, multivariate Cox-regression analysis showed PR-B to be the only independent prognostic marker for patient survival (HR 0.455, 95% CI 0.251-0.823, p=0.009) (Table 3).

**Table 3:** COX regression analysis for patient survival significant results are shown in bold

	**HR**	**95.0% CI lower upper**		**P-values**
**Grade**				**0.032**
Grade 1 vs. Grade 2	0.393	0.194	0.798	**0.010**
Grade 1 vs. Grade 3	0.727	0.452	1.169	0.188
**Stage**				**0.001**
FIGO I vs. FIGO II	0.113	0.022	0.569	**0.008**
FIGO I vs. FIGO III	0.333	0.057	1.929	0.220
FIGO I vs. FIGO IV	0.485	0.110	2.136	0.338
Age <50a vs. >50a	1.026	0.555	1.895	0.935
ER-α positive vs. ER-α negative	0.660	0.397	1.098	0.110
ER-β positive vs. ER-β negative	1.108	0.687	1.788	0.673
PR-A positive vs. PR-A negative	1.436	0.825	2.500	0.201
PR-B positive vs. PR-B negative	0.455	0.251	0.823	**0.009**

HR=hazard ratio, CI=confidence interval.

## Discussion

There are several studies describing ER and PR expression in breast, endometrial or prostate cancer [17,18,23]. In this study we assessed ER-α/-β and PR-A/-B tissue expression in OC by immunohistochemistry. OC tissue was positive for ER-α/-β in 31.4% and 60.1% and PR-A/-B in 36.2% and 33.8%, respectively. In literature there are comparable results, though a wide range of steroid receptor expression in OC is reported, namely 32–77% for ER and 26-43% for PR [19,24,25]. The great variability described for steroid hormone receptor expression in OC is probably attributable to different analytical methods, e.g. biochemical assays like the Dextran-coated charcoal method in former times and immunohistochemistry or reverse transcription–polymerase chain reaction (RT–PCR) today [20,24,26,27]. Even within one analytical method, results differ with regard to diverse cut-offs or scoring systems [28]. Moreover, several steroid receptor expression studies for OC have a limited tumor selection by restricting patient inclusion criteria to a certain histological subtype, grade or tumor stage [25,29]. But most important for the interpretation of ER and PR results in OC is the comparison of the identical steroid hormone receptor isoforms. As shown here in our study, tissue expression, receptor correlations and survival results for ER-α vs. ER-β or PR-A vs. PR-B differ in a wide range. Another limitation are the commercially available antibodies, which are suspected to have a variable affinity to steroid hormone receptors, potentially resulting in underestimation of receptor expression [30].

In vivo and in vitro studies reveal varying effects of estrogen and progesterone on OC cells. OC cell growth is mainly induced through ER-α, because at in vitro analysis only 17-beta-estradiol induced cell growth, a specific ER-α but not ER-β agonist [12]. Altogether, there is increasing evidence that ER-α is a tumor promoter acting on OC cell growth and proliferation, whereas ER-β has been described to have proapoptotic and antiproliferative effects [13,14]. Simpson et al. described PR-A to function as a transcriptional inhibitor of ER [15]. Kumar et al. found PR-B to be involved in cell differentiation [16].

Our clinical data confirm that there are interactions between ER and PR receptors as we observed several significant receptor correlations between ER-α, ER-β, PR-A and PR-B. Most importantly, PR-B, which we found to be of prognostic value in this study, appears to correlate significantly positively with ER-α and PR-A. This observation had previously also been made by others [31]. There was no correlation for PR-B and tumor stage, grade or histological subtype in this study. This finding is in accordance with a study of 322 OC tumor samples in which PR expression was not associated with tumor grade or clinical stage [24]. In contrast, others found a correlation with early clinical stage, less presence of ascites and better tumor differentiation [32]. Again, the comparison of study results is limited since there was no further differentiation of the ER and PR receptor isoforms.

Prognostic markers which could support a more individualized anti-OC therapy are scarce. Especially the prognostic roles of ER and PR have been discussed controversially. In this study, PR-B has been identified as an independent prognostic marker for OC patient survival beside tumor grade and stage. Moreover, ER-α positive and PR-B positive OC had a favorable outcome. In contrast, ER-β negative OC had a better survival. Our data are in accordance with studies by others who also found higher PR status [29], increased ER or combined ER/PR positive tumors to be associated with a favourable patient outcome [20,24,26,27]. Akhahira et al. examined the two isoforms PR-A and PR-B and ER-α by immunohistochemistry (107 cases) and reverse transcription-polymerase chain reaction (RT-PCR; 16 cases) [31]. They observed PR-B to be the only analyzed hormone receptor with prognostic relevance on multivariate analysis [31]. This finding is in agreement with our data presented here. Nonetheless, there are also studies showing no impact of ER or PR expression on patient survival [28,33].

In this study, a negative correlation of PR-A and LH-R expression was noted. Interestingly, our previously published data showed a prognostic value of LH-R in this patient group [34]. Since we only observed a prognostic value of PR-B but not of PR-A expression, this might also be attributable to PR-A and LH-R interactions.

Clinical study results using anti-estrogen or aromatase inhibitor therapy were often disappointing. Nearly all clinical studies included patients with all OC subtypes and histological grades or patients who were extensively pretreated with chemotherapy or had bulky disease [35-37]. Reported response rates to tamoxifen range between 0 and 56% [35,38,39]. In a recently published review on tamoxifen studies in OC, tamoxifen achieved objective overall response rates of 10% and stable disease rates of 32% [37]. Significantly different response rates to tamoxifen have been observed with regard to histological subtypes [36]. Altogether, it seems that study results concerning anti-hormone therapy should be stratified with regard to ER-α, ER-β, PR-A and RP-B expression since receptor isoforms seem to have varying functions in vivo and in vitro and therefore have different relevance for patient survival.

## Conclusions

To our knowledge, this study is the only work assessing the expression of the four isoforms ER-alpha, ER-beta, PR-A and PR-B receptor and patient survival in a large series of OC patients. ER-α/-β and PR-A/-B are frequently expressed in OC with a certain variability relating to histological subtype, grade and stage. Univariate analysis indicated a favorable outcome for ER-α positive and PR-B positive OC, while multivariate analysis showed PR-B to be the only independent prognostic marker for patient survival. The more specified analysis of steroid receptor expression, e.g. in combination with other newly identified prognostic markers like the LH/HCG receptor [34], could assist in choosing a more individualized and hopefully more effective OC therapy for certain patients.

## Competing interests

All authors declare to have no financial or non-financial competing interests. There is no funding source to be disclosed.

## Authors’ contributions

ML and TL have made substantial contributions to conception, design and acquisition of data. SH, ND and DM have made substantial contributions to analysis and interpretation of data. IH has been involved in drafting the manuscript and revising it critically for important intellectual content. KF and UJ have given final approval of the version to be published. In addition, KF and UJ have made substantial contributions to conception and design of the study. All authors read and approved the final manuscript.

## Pre-publication history

The pre-publication history for this paper can be accessed here:

http://www.biomedcentral.com/1471-2407/12/553/prepub
